# Chemical activation of divergent protein tyrosine phosphatase domains with cyanine-based biarsenicals

**DOI:** 10.1038/s41598-019-52002-1

**Published:** 2019-11-06

**Authors:** Bailey A. Plaman, Wai Cheung Chan, Anthony C. Bishop

**Affiliations:** 1 Amherst College, Department of Chemistry, Amherst, Massachusetts 01002 USA; 2 Present Address: Dana-Farber Cancer Institute, Department of Cancer Biology, Boston, MA 02215 USA

**Keywords:** Chemical tools, Enzymes

## Abstract

Strategies for the direct chemical activation of specific signaling proteins could provide powerful tools for interrogating cellular signal transduction. However, targeted protein activation is chemically challenging, and few broadly applicable activation strategies for signaling enzymes have been developed. Here we report that classical protein tyrosine phosphatase (PTP) domains from multiple subfamilies can be systematically sensitized to target-specific activation by the cyanine-based biarsenical compounds AsCy3 and AsCy5. Engineering of the activatable PTPs (*act*PTPs) is achieved by the introduction of three cysteine residues within a conserved loop of the PTP domain, and the positions of the sensitizing mutations are readily identifiable from primary sequence alignments. In the current study we have generated and characterized *act*PTP domains from three different subfamilies of both receptor and non-receptor PTPs. Biarsenical-induced stimulation of the *act*PTPs is rapid and dose-dependent, and is operative with both purified enzymes and complex proteomic mixtures. Our results suggest that a substantial fraction of the classical PTP family will be compatible with the *act*-engineering approach, which provides a novel chemical-biological tool for the control of PTP activity and the study of PTP function.

## Introduction

Protein tyrosine phosphatases (PTPs) catalyze the dephosphorylation of phosphotyrosine, a key reaction for the control of mammalian signal transduction^1^. Misregulated PTP activity has been implicated as causative in many human diseases, including leukemia, solid tumors, diabetes, and autoimmune disorders^2–6^. Targeted strategies that allow for the direct chemical activation of individual PTPs could prove beneficial, both for investigating the roles of PTP activities in specific signaling pathways and for validating PTPs as therapeutic targets for small-molecule activators^7^.

Enzyme activators, however, are generally much more difficult to develop than enzyme inhibitors, as most enzymes do not contain well characterized allosteric-activation sites^8–12^. These general challenges also apply to PTPs specifically; very few small-molecule PTP activators are known, and the ones that have been reported exhibit limited potency and/or selectivity^12–14^. For example, the compounds spermidine and mitoxantrone have been identified as weak activators of T-cell PTP (TCPTP), but are also known to have many non-TCPTP-specific activities^13–16^. Promisingly, Tautermann *et al*. recently identified BI-0314, a small-molecule activator of PTPN5 that interacts with a previously uncharacterized site on the enzyme’s phosphatase domain^12^. BI-0314, however, suffers from limited potency, augmenting PTPN5’s activity by only 60% at a compound concentration of 500 μM^12^.

One possibility for avoiding the difficulties inherent in discovering activators of wild-type enzymes is to engineer activator sensitivity into target enzymes of interest^10^. Proteins that have been suitably engineered, either through fusion with other protein domains or through site-directed mutagenesis, can potentially be activated using optogenetics tools^17–19^, chemical inducers of dimerization^20,21^, or chemical rescue^22–29^. By and large, these activation approaches utilize light or small molecules to convert a target engineered protein from an “off” state to an “on” state.

By contrast, we have attempted to develop methods for engineering activatable PTPs that retain wild-type-like enzymatic activities and regulatory-control mechanisms until a small-molecule activator is administered^30,31^. In this vein, we recently reported that the phosphatase PTP1B can be rendered activatable by targeting the enzyme’s WPD loop, a structural feature that is conserved among classical PTPs^31^. We showed that the biarsenical compound AsCy3^32^ can be used to chemically activate a mutant of PTP1B (*act*PTP1B) that contains three cysteine point mutations in its WPD loop. We also showed that the *act*-engineering approach can be applied to PTP1B’s closest homolog, TCPTP, which shares 72% PTP-domain identity with PTP1B^31^.

In the current study we demonstrate that the tri-cysteine *act*-mutation motif, which was developed on PTP1B, can be applied to divergent subfamilies of classical PTPs. Guided by PTP-domain primary-sequence alignments, we show that WPD-loop engineering generates three new PTP-domain constructs—*act*HePTP, *act*PTPκ, and *act*SHP2—whose enzymatic activities are potently augmented by administration of the cyanine-based biarsenicals AsCy3 and/or AsCy5. These novel *act*PTPs, which come from distinct PTP subfamilies, include both receptor and non-receptor PTP domains and do not share a high degree of homology, neither with each other nor with PTP1B/TCPTP. Our results therefore suggest that a substantial fraction of the classical PTP family can be readily sensitized to activation by AsCy3/AsCy5 and that *act*PTPs may constitute a widely applicable tool for the control of PTP activity and the study of PTP function.

## Results and Discussion

### Design of *act*PTPs

**Figure 1:**
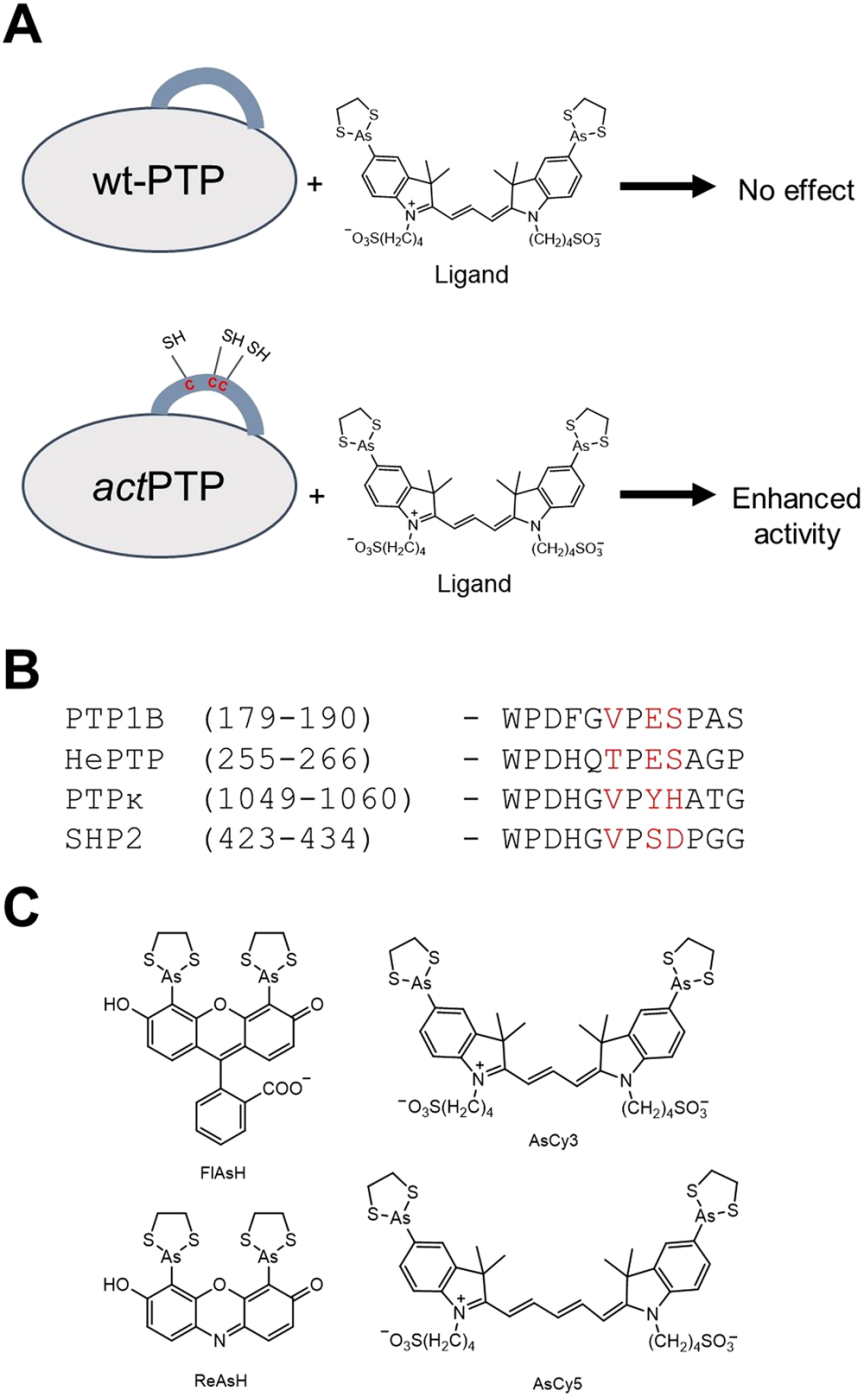
Design of chemically activatable (*act*) PTPs. (**A**) In *act*PTPs, three cysteines are engineered at non-conserved residues within the WPD loop to create a tricysteine motif. Engagement of a biarsenical ligand with the engineered binding site on the WPD loop enhances *act*PTP activity while wild-type activity is unaffected. (**B**) Primary sequence alignment of the WPD loops of wild-type PTPs. In the WPD loop sequence of *act*PTPs, cysteines replace the positions colored in red. (**C**) Chemical structures of four biarsenicals used to target *act*PTPs.

We have previously shown that the biarsenical compound AsCy3, which does not affect the activities of wild-type PTP1B or TCPTP, successfully binds to and activates the tri-cysteine mutants *act*PTP1B and *act*TCPTP (Fig. 1A)^31^. To test the scope of the *act*-engineering approach across the classical PTP family, we selected three biologically important PTPs from distinct subfamilies, including both receptor-like (R) and non-transmembrane (NT) classical PTPs: hematopoietic PTP (HePTP), PTPkappa (PTPκ), and Src-homology-2-domain-containing PTP 2 (SHP2)^33^. HePTP (subtype R7, 38% PTP-domain identity with PTP1B) is expressed in white blood cells, negatively regulates T-cell activation and proliferation, and is overexpressed in some preleukemic myeloproliferative diseases^34^. PTPκ (subtype R2A, 37% PTP-domain identity with PTP1B) is a receptor PTP that has been implicated as a breast-cancer suppressor^35^. Downregulation of PTPκ has also been linked to melanoma, lung cancer, and prostate cancer^36^. SHP2 (subfamily NT2, 39% PTP-domain identity with PTP1B) provides another example of the strong connection between misregulation of PTP activity and human disease, as germline SHP2 mutations cause Noonan and LEOPARD syndromes^37–39^ and activating somatic SHP2 mutations are the most common cause of sporadic juvenile myelomonocytic leukemia^40,41^.

To design putatively activatable mutants of HePTP, PTPκ, and SHP2, we aligned each enzyme’s WPD-loop sequence with that of PTP1B and identified the amino-acid residues that correspond to the cysteine-mutation sites of *act*PTP1B (V184, E186, and S187 of wild-type PTP1B, Fig. 1B). Site-directed mutagenesis was used to place three cysteine residues at the corresponding WPD-loop positions to generate *act*HePTP and *act*PTPκ. To design *act*SHP2, we coupled the analogous tri-cysteine WPD loop mutation with the removal of a non-conserved cysteine (C333) at a different site on the enzyme’s PTP domain, due to the previous observation that C333 renders SHP2 sensitive to inhibition by biarsenical compounds^42^. To avoid potential competition between biarsenical-induced activation and inhibition in the tri-cysteine engineered SHP2, we mutated C333 to proline, the residue that is found at the corresponding position in the vast majority of human classical PTP domains^33^. The three engineered proteins, *act*HePTP, *act*PTPκ, and *act*SHP2, expressed well in *Escherichia coli* and were isolated at purity levels indistinguishable from those of their wild-type counterparts.

### Identification of AsCy5 as an activator of *act*HePTP

**Figure 2:**
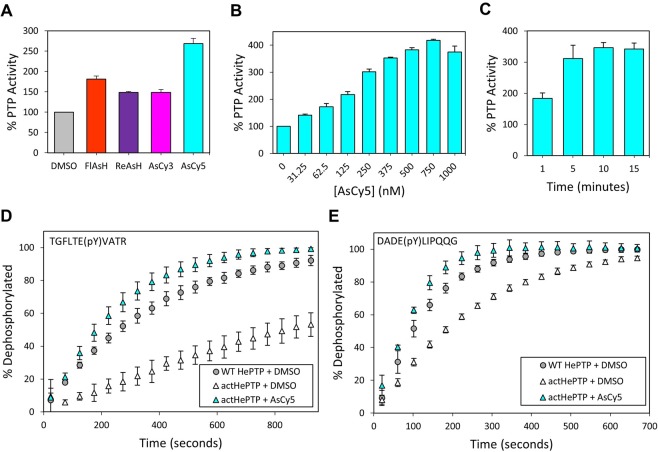
a*ct*HePTP is strongly activated by AsCy5. (**A**) PTP activity of *act*HePTP (200 nM) was measured with *p*NPP (1.5625 mM, quenched assay) in the absence (DMSO) or presence of the indicated biarsenicals (500 nM) after 120-minute pre-incubations. (**B**) Dose dependence of *act*HePTP activation. PTP activity of *act*HePTP (200 nM) was measured with *p*NPP (1.5625 mM, quenched assay) in the absence (DMSO) or presence of the indicated concentrations of AsCy5 after 20-min pre-incubations. (**C**) Time dependence of *act*HePTP activation. PTP reaction rates of *act*HePTP (200 nM) were measured with *p*NPP (2 mM, continuous assay) in the absence (DMSO) or presence of AsCy5 (625 nM) after indicated pre-incubation times. (**D**) The activity of wt-HePTP and *act*HePTP (2 µM) was measured with the phosphopeptide TGFLTEpYVATR (200 µM) as a substrate after 20-min of pre-incubation in the absence (DMSO) or presence of AsCy5 (4 µM). (**E**) The activity of wt-HePTP and *act*HePTP (2 µM) was measured with the phosphopeptide DADEpYLIPQQG (100 µM) as a substrate after 20-min of pre-incubation in the absence (DMSO) or presence of AsCy5 (4 µM).

As a first test of the possibility of extending the *act*PTP approach beyond the PTP1B/TCPTP subfamily (NT1) we selected *act*HePTP. To determine if *act*HePTP is targetable by biarsenicals, we incubated the purified enzyme with four commercially available compounds: FlAsH, ReAsH, AsCy3, and AsCy5 (Fig. 1C). Activity assays using the substrate *para*-nitrophenylphosphate (*p*NPP) revealed that all four biarsenicals induce substantial activation of *act*HePTP (Fig. 2A). In contrast to previous studies with *act*PTP1B and *act*TCPTP, on which AsCy3 was the strongest activator^31^, we found that AsCy5, a compound with a larger interarsenical distance, was the most effective activator of *act*HePTP. Pre-incubation with 500 nM AsCy5 gave rise to an approximately 175% increase in *act*HePTP’s dephosphorylation rate (Fig. 2A), but had no significant effect on the activity of wild-type HePTP (Supplementary Fig. S1A). These results demonstrate that the tri-cysteine WPD-loop mutation strategy can be extended beyond the NT1 subfamily of PTPs, and that screening a panel of biarsenicals with varying interarsenical distances can be useful for optimizing the efficacy of biarsenical-induced activation when applying the strategy to new PTP subfamilies.

### Characterization of AsCy5-induced activation of *act*HePTP

We next investigated the dose-dependence of AsCy5-induced activation of *act*HePTP. Following incubation of *act*HePTP (200 nM) with various concentrations of AsCy5, we observed potent dose-dependent activation that plateaued at approximately 750 nM AsCy5 and four-fold activation (Fig. 2B). At lower concentrations, relative activity increased steadily with increasing AsCy5; even the lowest concentration tested (31.25 nM) produced approximately 150% activity compared to the vehicle-only control. The remarkable potency of AsCy5-mediated activation is evidenced by the low compound concentration necessary to reach half maximum activation (AC_50_ ≈ 200 nM AsCy5), which approaches the assay’s concentration of enzyme in solution (200 nM *act*HePTP).

To assess the rate at which AsCy5 exerts its effect on *act*HePTP, we next measured the time dependence of activation. We incubated *act*HePTP with AsCy5 for various time periods and then tested for activity (Fig. 2C). AsCy5-induced activation was rapid, with nearly two-fold activation observed after only one minute of pre-incubation, and the assay’s maximum activation was observed within ten minutes.

We next compared the kinetic parameters of the activated *act*HePTP with those of the non-treated enzyme (Table 1 and Supplementary Fig. S2). We also directly compared the activity of untreated *act*HePTP with that of wild-type HePTP to assess the potential effect of the cysteine mutations on HePTP’s inherent enzymatic activity. We found that catalytic efficiency (*k*_cat_/*K*_M_) of *act*HePTP in the absence of biarsenical was roughly comparable to that of wild-type (Table 1). Although the catalytic rate constant (*k*_cat_) of untreated *act*HePTP was slightly lower than that of wild-type HePTP, the small reduction in *k*_cat_ was offset by a comparable reduction in Michaelis constant (*K*_M_). In the presence of AsCy5, the catalytic efficiency of *act*HePTP increased dramatically, with a 1.7-fold increase in *k*_cat_ and a 3.5-fold decrease in *K*_M_ combining for a six-fold increase in *k*_cat_/*K*_M_. It follows from the data described above that AsCy5-treated *act*HePTP dephosphorylates *p*NPP much more efficiently than does wild-type HePTP, predominantly due to a 5.6-fold decrease in *K*_M_ (Table 1). To our knowledge, AsCy5-treated *act*HePTP represents the first small-molecule modulator that is capable of increasing HePTP activity above that of the wild-type enzyme.

**Table 1:** Kinetic constants of wild-type (wt) HePTP, *act*HePTP, and AsCy5-treated (750 nM) *act*HePTP assayed with *p*NPP.

Enzyme	*k*_*cat*_ [s^−1^]	*K*_M_ [mM]	*k*_*cat*_*/K*_M_ [mM^−1^s^−1^]
wt HePTP + DMSO	2.0 ± 0.2	5.6 ± 0.8	0.36 ± 0.06
*act*HePTP + DMSO	1.40 ± 0.02	3.5 ± 0.1	0.40 ± 0.01
*act*HePTP + AsCy5	2.4 ± 0.1	1.0 ± 0.1	2.4 ± 0.3

### Activity of *act*HePTP with phosphopeptide substrates

For our engineered activation approach to be useful in the exploration of cellular pathways involving HePTP, *act*HePTP must be able to act on physiological substrates of HePTP, and AsCy5 must accentuate *act*HePTP activity regardless of substrate. To test both *act*HePTP’s ability to dephosphorylate a HePTP substrate and the substrate-dependence of AsCy5-induced activation of *act*HePTP, we measured the enzyme activity on the phosphopeptide TGFLTE(pY)VATR, which corresponds with a phosphorylation site on the mitogen-activated protein kinase 1 (MAPK1), a physiological substrate of HePTP^43^.

In the absence of AsCy5, *act*HePTP dephosphorylates TGFLTE(pY)VATR at a rate that is notably lower than that of wild-type HePTP, but addition of AsCy5 induces activation of *act*HePTP slightly above wild-type levels (Fig. 2D). The augmentation of *act*HePTP’s phosphopeptide dephosphorylation rate beyond that of wild-type HePTP is modest in comparison to the activation observed with *p*NPP as a substrate. However, these data importantly demonstrate that *act*HePTP is both active and activatable in the dephosphorylation of a model peptide based on the parent enzyme’s physiological substrate.

To determine whether activation levels are consistent among varying phosphopeptides, we also measured the effect of AsCy5 on *act*HePTP activity on the phosphopeptide DADE(pY)LIPQQG, which corresponds to an autophosphorylation site on the epidermal growth factor receptor (EGFR), a physiological substrate of PTP1B^44^. In line with our observation on TGFLTE(pY)VATR, *act*HePTP dephosphorylates DADE(pY)LIPQQG at a rate that is notably lower than that of wild-type HePTP in the absence of AsCy5, but addition of AsCy5 induces activation of *act*HePTP slightly beyond wild-type levels (Fig. 2E). Broadly, the *act*HePTP AsCy5-induced activation results were largely consistent between the two phosphopeptides tested, demonstrating that AsCy5 is capable of direct chemical activation of *act*HePTP on a variety of substrates.

### Targeting *act*HePTP with AsCy5 in complex proteomes

**Figure 3:**
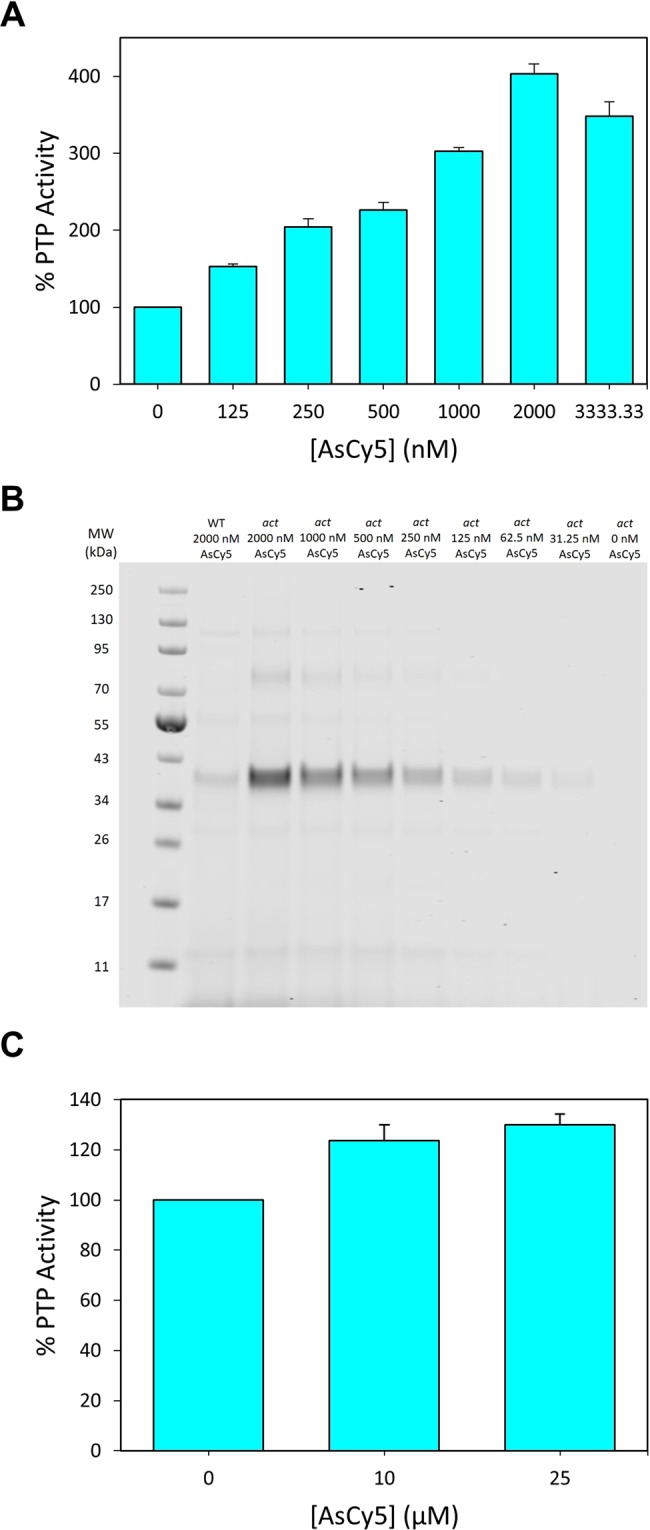
AsCy5 targets and activates *act*HePTP in complex proteomes and cells. (**A**) Activation of *act*HePTP in cell lysates. Clarified lysates (0.125 mg/mL) from *E. coli* expressing wt-HePTP or *act*HePTP were incubated with the indicated concentrations of AsCy5 for 60 minutes, then assayed for PTP activity with *p*NPP (1.5 mM, quenched assay). (**B**) Visualization of targeted proteins from AsCy5-treated cell lysates. Clarified lysates (0.085 mg/mL) expressing *act*HePTP or wt-HePTP were incubated with the indicated concentrations of AsCy5 for 15 minutes. The resulting solutions were diluted by a factor of 25, and 15 µL of each dilution were separated by SDS-PAGE. AsCy5-bound proteins were visualized using near infrared illumination. An uncropped image of the gel is presented in Supplementary Fig. S8. (**C**) Activation of *act*HePTP in intact cells. *E. coli* cells expressing either wild-type HePTP (wt) or *act*HePTP were incubated with the indicated concentrations of AsCy5 for 2 hours, washed thoroughly, and lysed. PTP activities of the resulting lysates were measured with *p*NPP (2 mM, quenched assay).

We next asked whether *act*HePTP could be targeted for direct chemical activation in the presence of a complex proteome. To do so, we measured the dose-dependence of AsCy5-induced activation of *act*HePTP in a cell lysate derived from *act*HePTP-expressing *Escherichia coli*. (Because the *E*. *coli* genome does not encode PTPs, total PTP activity of a crude *E. coli* lysate can be interpreted as representative of the activity of an overexpressed mammalian PTP.) After incubating lysates from *act*HePTP-expressing cells with various concentrations of AsCy5 (or vehicle control), we assayed each mixture for PTP activity with *p*NPP (Fig. 3A). Similar to results on purified *act*HePTP, AsCy5-induced activation of *act*HePTP in a crude lysate was dose-dependent and robust, although activation in lysates required moderately higher AsCy5 concentrations (AC_50_ ≈ 500 nM) than the activation of purified *act*HePTP (AC_50_ ≈ 200 nM).

To investigate whether the need for higher AsCy5 concentrations was due to off-target binding within the crude cellular lysate, we took advantage of AsCy5’s inherent fluorescent properties. After incubating crude lysates with various concentrations of AsCy5, we separated the lysate proteins by SDS-PAGE and visualized AsCy5-bound proteins with near-IR light. Even at the highest AsCy3 concentration (2000 nM), the lane derived from an AsCy5-treated wild-type HePTP-expressing lysate displayed no strong bands, whereas lanes derived from AsCy5-treated *act*HePTP-expressing lysates each displayed a strong band that is found at a position corresponding to the molecular weight of HePTP (35 kDa, Fig. 3B). This band diminishes in intensity with decreasing concentrations of AsCy5, consistent with the observed dose-dependent activation. These results suggest that AsCy5 engages with *act*HePTP with high specificity, even in a complex proteomic environment.

The ability of AsCy5 to cross membranes has not been investigated, but the compound’s cell permeability is presumably hindered due to its two negatively charged sulfonate moieties, much like the structurally similar AsCy3 (Fig. 1C)^45^. However, we previously found that high concentrations of AsCy3 can be used to target *act*PTP1B in intact cells^31^, leading us to investigate AsCy5’s ability to activate *act*HePTP in *E. coli*. We incubated *act*HePTP-expressing cell suspensions with various concentrations of AsCy5 and washed away free compound. After lysis and clarification, the resulting solutions were tested for PTP activity (Fig. 3C). The data suggest that AsCy5 has very limited cellular permeability, at least in bacteria, as the observed AsCy5-induced *act*HePTP activation after in-cell treatment was not nearly as potent or as robust as observed on purified enzyme and in membrane-free proteomic mixtures. It is likely that targeting of *act*HePTP in mammalian cells would require the development of AsCy5 analogs that lack the parent compound’s sulfonate groups.

### AsCy5-induced activation of *act*PTPκ

**Figure 4:**
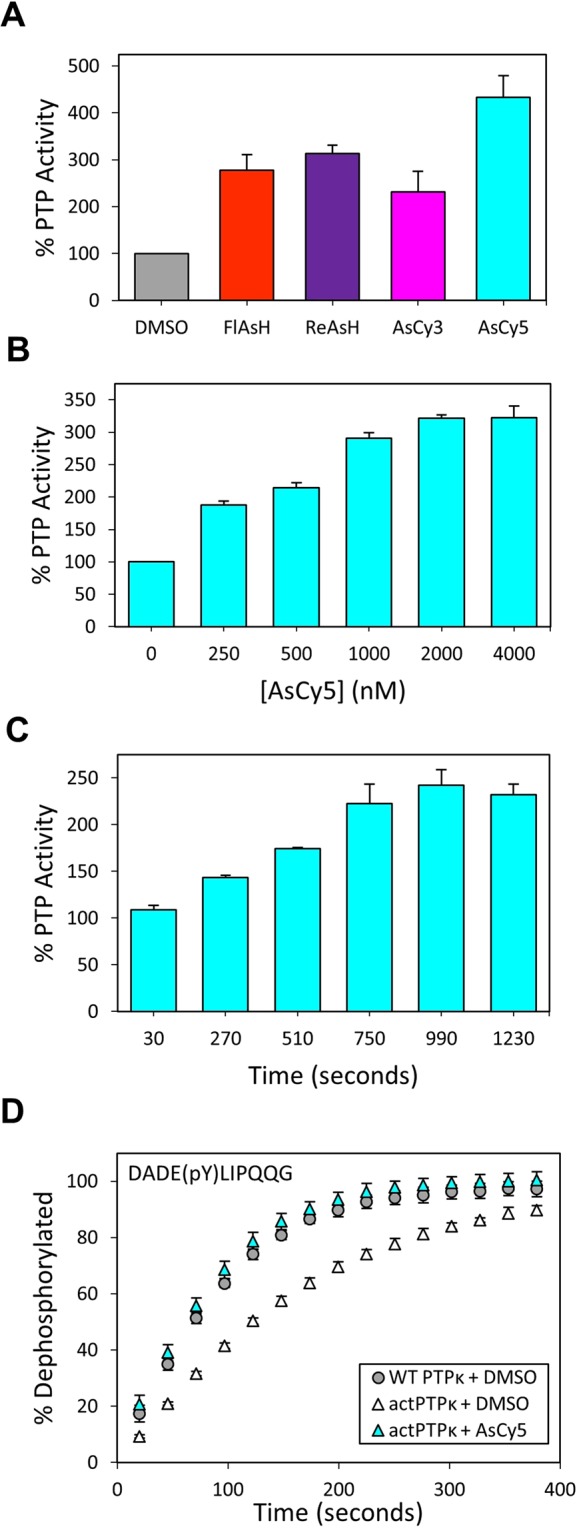
ac*t*PTPκ is strongly activated by AsCy5. (**A**) PTP activity of *act*PTPκ (400 nM) was measured with *p*NPP (2 mM, quenched assay) in the absence (DMSO) or presence of the indicated biarsenicals (2 µM) after 60-minute pre-incubations. (**B**) Dose dependence of *act*PTPκ activation. PTP activity of *act*PTPκ (400 nM) was measured with *p*NPP (2 mM, quenched assay) in the absence (DMSO) or presence of the indicated concentrations of AsCy5 after 30-min pre-incubations. (**C**) Time dependence of *act*PTPκ activation. PTP reaction rates of *act*PTPκ (400 nM) were measured with *p*NPP (2 mM, continuous assay) in the absence (DMSO) or presence of AsCy5 (2 µM) after indicated pre-incubation times. (**D**) The activity of wt-PTPκ and *act*PTPκ (100 nM) was measured with the phosphopeptide DADEpYLIPQQG (100 µM) as a substrate after 30-min of pre-incubation in the absence (DMSO) or presence of AsCy5 (4 µM).

We further explored the generalizability of our activation approach by extending our studies to the putatively activatable *act*PTPκ. We investigated the potential for biarsenical-induced activation of *act*PTPκ by incubating the enzyme with FlAsH, ReAsH, AsCy3, and AsCy5. Like *act*HePTP, *act*PTPκ activity was most robustly augmented by AsCy5 (approximately 425% activity), while the effects of FlAsH, ReAsH, and AsCy3 were more modest (Fig. 4A). We therefore selected AsCy5 for further characterization of *act*PTPκ activation.

To characterize the dose-dependence of AsCy5-mediated activation of *act*PTPκ we measured its activity following incubation with various concentrations of AsCy5. We observed dose-dependent activation of *act*PTPκ that reached AC_50_ at approximately 600 nM and plateaued at 2000 nM AsCy5 (Fig. 4B). Although the dose-dependent activation of *act*PTPκ appears to be 2–3-fold less potent than the activation of *act*HePTP (compare Fig. 4B with 2B), the apparent discrepancy is most likely due to the different enzyme concentrations used (actHePTP: 200 nM, *act*PTPκ: 400 nM), as AsCy5-induced activation of both enzymes approaches the theoretical potency limits of the assays.

To assess the rate at which AsCy5 binds to *act*PTPκ’s engineered tri-cysteine motif on the WPD loop, we measured the time dependence of activation. We incubated *act*PTPκ with AsCy5 for various time periods and then tested for activity (Fig. 4C). AsCy5-induced activation was rapid, with maximum activation observed after approximately sixteen minutes of pre-incubation.

Michaelis-Menten analysis of wild-type PTPκ and *act*PTPκ reveals that AsCy5-treated *act*PTPκ shows a five-fold increase in catalytic efficiency when compared to a vehicle-only *act*PTPκ control and a four-fold increase in catalytic efficiency when compared to wild-type PTPκ (Table 2 and Supplementary Fig. S3). As with *act*HePTP, this increase is due to a combination of an increase in *k*_cat_ and a decrease in *K*_M_. Additionally, AsCy5 successfully activates *act*PTPκ in the dephosphorylation of phosphopeptide DADE(pY)LIPQQG. The dephosphorylation of DADE(pY)LIPQQG by *act*PTPκ occurs at a rate that is notably lower than that of wild-type PTPκ in absence of AsCy5, but addition of AsCy5 induces activation of *act*PTPκ back to wild-type levels (Fig. 4D).

**Table 2:** Kinetic constants of wild-type (wt) PTPκ, *act*PTPκ, and AsCy5-treated (4 µM) *act*PTPκ assayed with *p*NPP.

Enzyme	*k*_*cat*_ [s^−1^]	*K*_M_ [mM]	*k*_*cat*_*/K*_M_ [mM^−1^s^−1^]
wt PTPκ + DMSO	1.49 ± 0.04	6.0 ± 0.5	0.26 ± 0.02
*act*PTPκ + DMSO	1.09 ± 0.04	5.3 ± 0.3	0.21 ± 0.01
*act*PTPκ + AsCy5	4.1 ± 0.2	3.9 ± 0.2	1.05 ± 0.08

**Figure 5:**
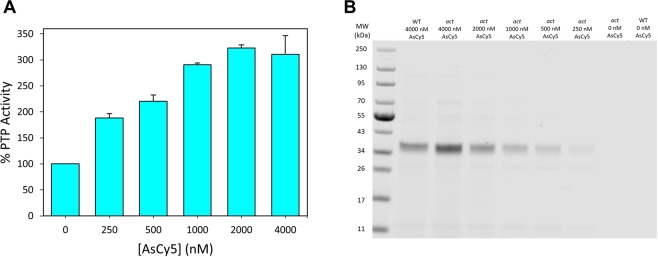
AsCy5 targets and activates *act*PTPκ in a complex cellular proteome. (**A**) Clarified lysates (0.125 mg/mL) from *E. coli* expressing wt-PTPκ or *act*PTPκ were incubated with the indicated concentrations of AsCy5 for 45 minutes, then assayed for PTP activity with *p*NPP (2 mM, quenched assay). (**B**) Visualization of targeted proteins from AsCy5-treated cell lysates. Clarified lysates (0.125 mg/mL) expressing *act*PTPκ or wt-PTPκ were incubated with the indicated concentrations of AsCy5 for 30 minutes. The resulting solutions were diluted by a factor of 75, and 15 µL of each dilution were separated by SDS-PAGE. AsCy5-bound proteins were visualized using near infrared illumination. An uncropped image of the gel is presented in Supplementary Fig. S8.

We next investigated AsCy5-induced activation of *act*PTPκ in *E. coli* lysates. The dose-dependent activation of *act*PTPκ in the proteomic mixture is remarkably similar to the dose-dependent activation of purified *act*PTPκ; maximum activity of *act*PTPκ within the crude lysate is approximately 325% at 2000 nM AsCy5 (Fig. 5A). Visualization of lysate proteins after SDS-PAGE separation and exposure to near-IR light shows one strong band in all lanes at a position that corresponds with the molecular weight of the PTPκ catalytic domain (34 kDa), with band intensity diminishing with decreasing AsCy5 concentration (Fig. 5B). Unexpectedly, we found that a high concentration of AsCy5 (4000 nM) also produced detectable labeling of the wild-type PTPκ catalytic domain (Fig. 5B), even though wild-type PTPκ contains no engineered tri-cysteine motif. Closer inspection of PTPκ’s primary and tertiary structure revealed that, in contrast to the other PTPs studies here, the C-terminal portion of PTPκ’s catalytic domain contains three cysteine residues in close proximity to one another (C1143, C1145, and C1153, Supplementary Fig. S4)^46^. This non-conserved cysteine-rich motif of wild-type PTPκ likely constitutes the AsCy5-binding site suggested by the data in Fig. 5B. Importantly, PTPκ’s naturally occurring cysteine-rich motif is not near the enzyme’s active site, and we found that the activity of wild-type PTPκ is neither activated nor significantly inhibited by AsCy5 (Supplementary Fig. S1B).

### AsCy3-induced activation of *act*SHP2

**Figure 6:**
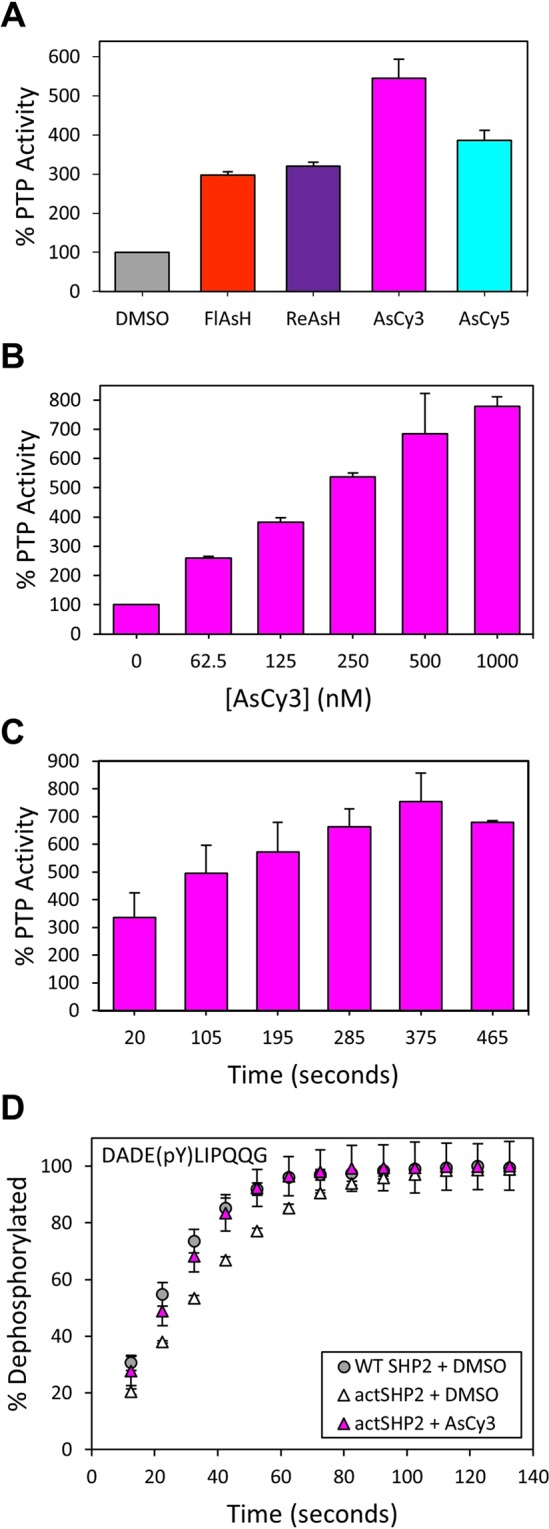
a*ct*SHP2 is strongly activated by AsCy3. (**A**) PTP activity of *act*SHP2 (50 nM) was measured with *p*NPP (2 mM, quenched assay) in the absence (DMSO) or presence of the indicated biarsenicals (1 µM) after 60-minute pre-incubations. (**B**) Dose dependence of *act*SHP2 activation. PTP activity of *act*SHP2 (50 nM) was measured with *p*NPP (2 mM, quenched assay) in the absence (DMSO) or presence of the indicated concentrations of AsCy3 after 60-min pre-incubations. (**C**) Time dependence of *act*SHP2 activation. PTP reaction rates of *act*SHP2 (50 nM) were measured with *p*NPP (2 mM, continuous assay) in the absence (DMSO) or presence of AsCy3 (500 nM) after indicated pre-incubation times. (**D**) The activity of wt-SHP2 and *act*SHP2 (50 nM) was measured with the phosphopeptide DADEpYLIPQQG (200 µM) as a substrate after 15-min of pre-incubation in the absence (DMSO) or presence of AsCy3 (500 nM).

We next investigated the biarsenical-induced activation of *act*SHP2, by measuring its activity after pre-incubation with FlAsH, ReAsH, AsCy3, and AsCy5 (Fig. 6A). Both cyanine-derived biarsenicals, AsCy3 and AsCy5, were more successful in activating *act*SHP2 than FlAsH and ReAsH. AsCy3 was the most robust activator (approximately 550% activity), and was selected for further characterization.

Upon testing the dose-dependence of AsCy3-mediated activation of *act*SHP2, we observed robust and potent activation that plateaued between 500 nM and 1000 nM AsCy3 at approximately 800% activity (Fig. 6B). We then incubated *act*SHP2 with AsCy3 for various time periods to assess the rate at which AsCy3 acts on the engineered tri-cysteine motif of the WPD loop (Fig. 6C). AsCy3 binds quickly, with over 300% activity observed at 20 seconds and maximum activation observed after approximately six minutes. Consistent with previous findings that the presence of the non-conserved cysteine C333 renders SHP2 sensitive to inhibition by biarsenicals, we found that wild-type shows some inhibition in the presence of 1000 nM AsCy3 (Supplementary Fig. S1C), validating the previously described engineering strategy of introducing the C333P mutation into *act*SHP2.

Michaelis-Menten analysis of wild-type SHP2 and *act*SHP2 reveals that the activity of *act*SHP2 is virtually indistinguishable from that of wild-type SHP2 in the absence of biarsenical. AsCy3-treated *act*SHP2 shows a remarkable ten-fold increase in catalytic efficiency due, again, to the combination of an increase in *k*_cat_ and a decrease in *K*_M_ (Table 3 and Supplementary Fig. S5). This AsCy3-induced activation of *act*SHP2 persists to a lesser extent in the desphosphorylation of phosphopeptide DADE(pY)LIPQQG. The dephosphorylation of phosphopeptide DADE(pY)LIPQQG by *act*SHP2 occurs at a slightly lower rate than dephosphorylation by wild-type SHP2 (Fig. 6D). Addition of AsCy3 restores the activity of *act*SHP2 back up to wild-type levels.

**Table 3:** Kinetic constants of wild-type (wt) SHP2, *act*SHP2, and AsCy3-treated (1 µM) *act*SHP2 assayed with *p*NPP.

Enzyme	*k*_*cat*_ [s^−1^]	*K*_M_[mM]	*k*_*cat*_*/K*_M_ [mM^−1^s^−1^]
wt SHP2 + DMSO	6.8 ± 0.5	4.6 ± 0.7	1.5 ± 0.3
*act*SHP2 + DMSO	6.5 ± 0.3	4.7 ± 0.4	1.4 ± 0.1
*act*SHP2 + AsCy3	28 ± 1	1.9 ± 0.3	15 ± 2

### Sensitivity of *act*PTPs can be governed by the residue preceding the *act* motif

**Figure 7:**
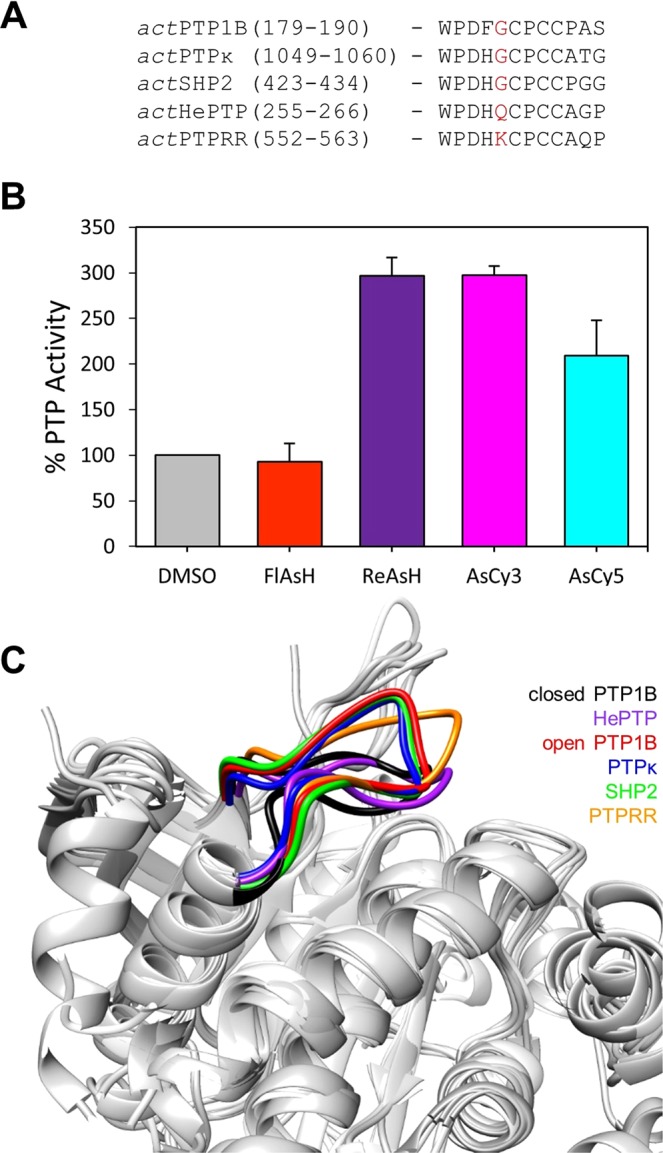
Design and characterization of *act*PTPRR. (**A**) Primary sequence alignment of the WPD loops of *act*PTP1B, *act*PTPκ. *act*SHP2, *act*HePTP, and *act*PTPRR. The position corresponding to K556 of PTPRR is highlighted in red. (**B**) PTP activity of *act*PTPRR (200 nM) was measured with *p*NPP (2 mM, quenched assay) in the absence (DMSO) or presence of the indicated biarsenicals (1 µM) after 60-minute pre-incubations. (**C**) Structural overlay of WPD loops. The WPD loops of PTP1B in the closed conformation (PDB ID: 1SUG, black)^48^, PTP1B in the open conformation (PDB ID: 2HNP, red)^49^, HePTP (PDB ID: 2A3K, purple)^50^, PTPκ (PDB ID: 2C7S, blue)^46^, SHP2 (PDB ID: 3B7O, green)^50^, and PTPRR (PDB ID: 2A8B, orange)^47^ are modeled using the UCSF Chimera software package.

To explore potential application of the *act-*engineering strategy across multiple members of a single PTP subfamily, we selected PTPRR, a second member of HePTP’s subfamily, R7^33^. Based on the *act*HePTP prototype, we generated the putatively activatable *act*PTPRR through introduction of a WPD-loop tricysteine motif (Fig. 7A). In contrast to previous examples of *ac*tPTPs, however, *act*PTPRR possesses catalytic activity that is notably reduced from that of wild-type PTPRR (Table 4 and Supplementary Fig. S6). We further found that, although *act*PTPRR is technically activatable by biarsenicals (approximately 300% activity with ReAsH and AsCy3, Fig. 7B), its biarsenical-induced rate enhancements are relatively modest when compared to its 13-fold defect in inherent catalytic efficiency (Table 4).

**Table 4:** Kinetic constants of wild-type (wt) PTPRR and *act*PTPRR, assayed with *p*NPP.

Enzyme	*k*_*cat*_ [s^−1^]	*K*_M_ [mM]	*k*_*cat*_*/K*_M_ [mM^−1^s^−1^]
wt PTPRR	3.61 ± 0.05	0.84 ± 0.07	4.4 ± 0.4
*act*PTPRR	0.90 ± 0.06	2.6 ± 0.4	0.35 ± 0.06

Inspection of *act*PTP WPD-loop sequences outside of the cysteine-mutation sites revealed a potential reason for the failure of the *act* strategy when applied to PTPRR. PTPRR contains a lysine residue at position 556, a position that lies adjacent to the first cysteine residue of the *act* motif and is occupied by glycine in the vast majority of human PTP domains (Fig. 7A)^33^. PTPRR is one of only two PTPs that have a lysine at this position, and the *act*PTPs that have been the most robustly responsive to biarsenicals in this and previous studies (*e.g*., *act*PTP1B, *act*TCPTP, *act*SHP2)^31^ all have glycine at the corresponding position. Additionally, x-ray crystallography experiments have previously shown that PTPRR’s WPD loop adopts an unusual intermediate (neither open nor closed) structure (Fig. 7C)^47^. Although K556’s role, if any, in favoring the intermediate WPD-loop structure is unknown, the sequence and structural idiosyncrasies of the PTPRR WPD loop, coupled with *act*PTPRR’s low responsiveness to biarsencials, led us to hypothesize that the presence of the PTP-consensus residue, glycine, at position 556 may be necessary for strong activation (>500%) of *act*PTPs.

**Figure 8:**
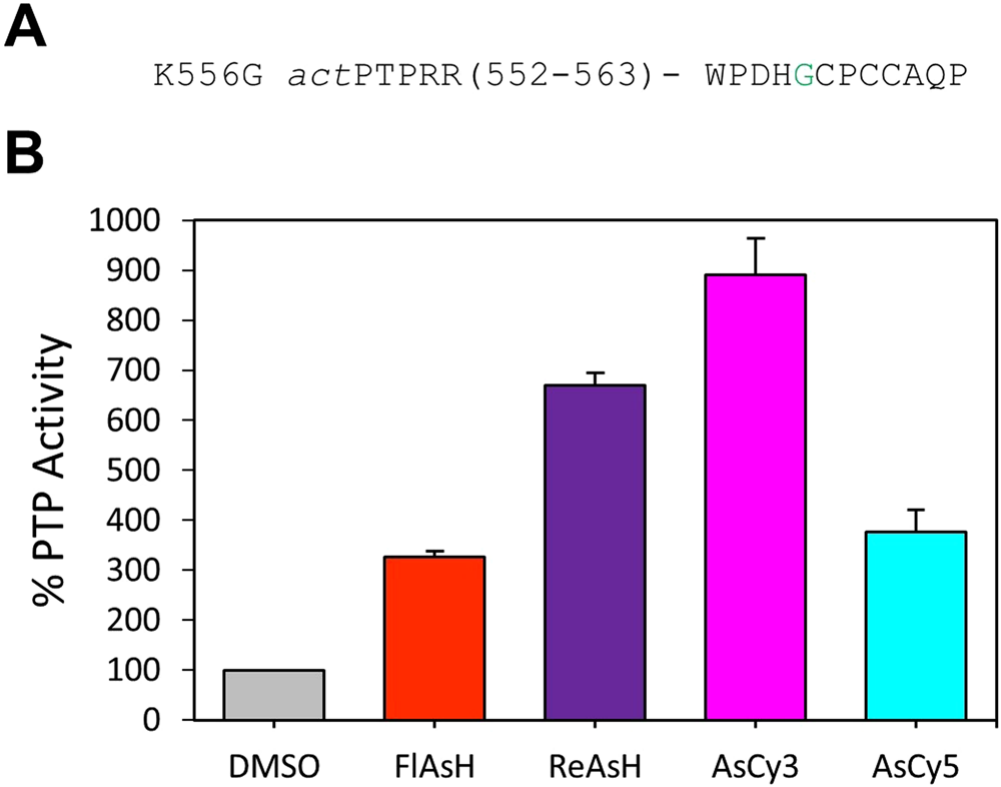
K556G *act*PTPRR is strongly activated by AsCy3. (**A**) Primary sequence of the WPD loop of K556G *act*PTPRR. The location of the glycine mutation is highlighted in green. (**B**) PTP activity of K556G *act*PTPRR (200 nM) was measured with *p*NPP (2 mM, quenched assay) in the absence (DMSO) or presence of the indicated biarsenicals (1 µM) after 60-minute pre-incubations.

To test this hypothesis we generated K556G *act*PTPRR (Fig. 8A) and tested its activity in the absence and presence of biarsenicals. Although the glycine mutation at position 556 did not rescue the inherent catalytic defect of *act*PTPRR, as shown by K556G *act*PTPRR’s low catalytic efficiency (Supplementary Fig. S7), activation of K556G *act*PTPRR with the biarsenical AsCy3 was found to be robust (Fig. 8B). The approximately 900% activity of K556G *act*PTPRR that is induced by AsCy3 is on par with, or exceeds, the strong activation observed on *act*PTPs that naturally contain glycine at this position (*act*PTP1B, *act*TCPTP, *act*SHP2, and *act*PTPκ)^31^. These results strongly suggest that the presence of the WPD-loop’s consensus glycine residue is essential for optimal biarsenical-induced activation of *act-*engineered PTPs, and that future applications of *act*PTP strategy will find the strongest success with PTPs that naturally possess the consensus residue. Since 31 out of 37 human classical PTPs do have glycine at this position, however, the “glycine criterion” constraint should not present a major impediment to the prospect of broadly applying the *act*PTP strategy.

## Conclusions

Although small-molecule-based protein activation strategies could provide powerful tools for probing cell-signaling pathways, few such methods have been described. Here we have shown that sequence-guided engineering of the WPD loop of classical PTPs provides a highly generalizable means to render PTPs activatable by the cyanine-based biarsenicals AsCy3 and AsCy5. We have shown that *act*PTPs can be systematically generated from various subfamilies of both receptor and non-receptor PTPs and that biarsenical-induced stimulation of *act*PTPs is rapid, dose-dependent, and potent, even in the context of complex proteomic mixtures. Although one specific WPD-loop sequence requirement for successful *act*PTP engineering was also outlined in this study, the limitation presented by this requirement is minor, as the majority of classical PTPs contain the key sequence element. In sum, our findings establish the broad generalizability of *act*PTP engineering and suggest that a substantial fraction of the classical PTP family will be compatible with the approach, which provides a novel tool for the small-molecule-based induction of PTP activity and the study of PTP function.

## Methods

### General and materials

“% PTP Activity” is defined as enzymatic reaction rate in the presence of a biarsenical as a percentage of the rate of a corresponding DMSO control (100%). Error bars and “±” values represent the standard deviation of at least three independent measurements. Biarsenicals were obtained commercially and used without further purification.

### Cloning and mutagenesis of PTP-encoding genes

The plasmid encoding the His_6_-tagged catalytic domains of SHP2 has been previously described^42^. Plasmids for expression of the His_6_-tagged catalytic domains of HePTP (pET-HePTP, residues 65–360), PTPκ (pDK002, residues 870–1154), and PTPRR (pBAP004, residues 373–657) were obtained commercially. Site-directed mutations were introduced using the Quikchange mutagenesis kit. Putatively activatable mutants (*act*PTPs) are defined as follows: *act*HePTP: T260C/E262C/S263C HePTP; *act*PTPκ: V1054C/Y1056C/H1057C PTPκ; *act*SHP2: C333P/V428C/S430C/D431C SHP2; *act*PTPRR: T557C/D559C/S560C PTPRR.

### Protein expression and purification

Protein expressions and purifications were performed essentially as previously described for other His_6_-tagged PTPs^42^. After purification, proteins were exchanged into storage buffer (50 mM 3,3-dimethylglutarate at pH 7.0, 1 mM EDTA, supplemented with 1 mM TCEP), flash-frozen, and stored at −80 °C as previously described^31^.

### Phosphatase activity assays with purified enzymes

#### Quenched phosphatase activity assay using *p*NPP

Quenched activity assays using *p*NPP as substrate were performed as previously described^31^. Briefly, PTP reactions were carried out in PTP buffer (50 mM 3,3-dimethylglutarate at pH 7.0, 1 mM EDTA, 50 mM NaCl) containing enzyme (varying concentrations: see figures), 1% DMSO or biarsenical in DMSO solutions (varying concentrations), and *p*NPP (added after varying biarsenical incubation times and at varying concentrations) at 22 °C. Reactions were quenched with base and the absorbances (405 nm) were measured.

#### Continuous phosphatase activity assay with *p*NPP

Continuous activity assays with *p*NPP were carried out as previously described^31^. Briefly, after pre-incubations of varying times (see figures) in PTP buffer (see above), reactions were started by the addition of *p*NPP (at varying concentrations) to solutions containing PTP (at varying concentrations) and biarsenical (at varying concentrations) or DMSO. The absorbances (405 nm) of the resulting solutions were measured continuously.

#### Continuous phosphatase activity assay with phosphopeptide substrate

PTP kinetic assays with phosphopeptides DADEpYLIPQQC and TGFLTEpYVATR were carried out by measuring increasing absorbance at 282 nm, essentially as described^31^. Assays were performed at 22 °C in peptide buffer (50 mM 3,3-dimethylglutarate at pH 7.0, 50 mM NaCl) with biarsenical (varying concentrations) or DMSO (1%) and the appropriate PTP (varying concentrations). Reactions were started by the addition of phosphopeptide (varying concentrations: see figures).

#### PTP assays with biarsenical-treated crude cell lysates

PTP assays with biarsenical-treated crude cell lysates were performed essentially as previously described^31^. *E. coli* overexpressing the appropriate PTP were grown and lysed. Protein concentrations of the clarified lysates were normalized, and lysate PTP activities were assessed as described above (quenched *p*NPP assay). Total protein concentration in the PTP assays varied between PTPs (see figures).

#### PTP assays with biarsenical-treated *E. coli* cells

PTP assays with biarsenical-treated *E. coli* cells were performed essentially as previously described^31^. Briefly, BL21(DE3) *E. coli* cells overexpressing the appropriate PTP were grown and harvested, resuspended, and incubated with either biarsenical (varying concentrations) or DMSO for 2 hours. After incubation, the cells were pelleted, washed and lysed. PTP activity in the clarified lysates was determined as described above (quenched *p*NPP assay).

### SDS-PAGE and visualization of biarsenical-binding proteins

Lysates from *E. coli* overexpressing the appropriate PTP were prepared as described above. Biarsenicals (varying concentrations) were added to lysate solutions (total protein concentrations vary: see figures). After incubation, 15 µL of diluted lysate proteins were separated by SDS-PAGE gel and visualized with a focus offset of 1.5 mm using the 700 nm laser of the Li-Cor Odyssey CLx Near-Infrared Imaging System. PageRuler Prestained NIR Protein Ladder was loaded for the determination of protein molecular weights.

## Supplementary information

Supplementary Information

## Data Availability

Supporting data is available from the authors.
