# Supplementary material for: Chemical activation of divergent protein tyrosine phosphatase domains with cyanine-based biarsenicals
Source: Sci Rep. 2019 Nov 6;9:16148. doi: 10.1038/s41598-019-52002-1 (PMC6834593; doi:10.1038/s41598-019-52002-1)
Supplement: Supplementary file 1 — Supplementary Information [file 41598_2019_52002_MOESM1_ESM.pdf]

Supplementary Information for:

Chemical activation of divergent protein tyrosine phosphatase domains with cyanine-based biarsenicals

Bailey A. Plaman,[1] Wai Cheung Chan,[1,2] and Anthony C. Bishop[1,*]

[1] Amherst College, Department of Chemistry, Amherst, Massachusetts 01002, USA

[2] Current Address: Dana-Farber Cancer Institute, Department of Cancer Biology, Boston, MA 02215, USA

* Correspondence and requests for materials should be addressed to A.C.B. (email: acbishop@amherst.edu)

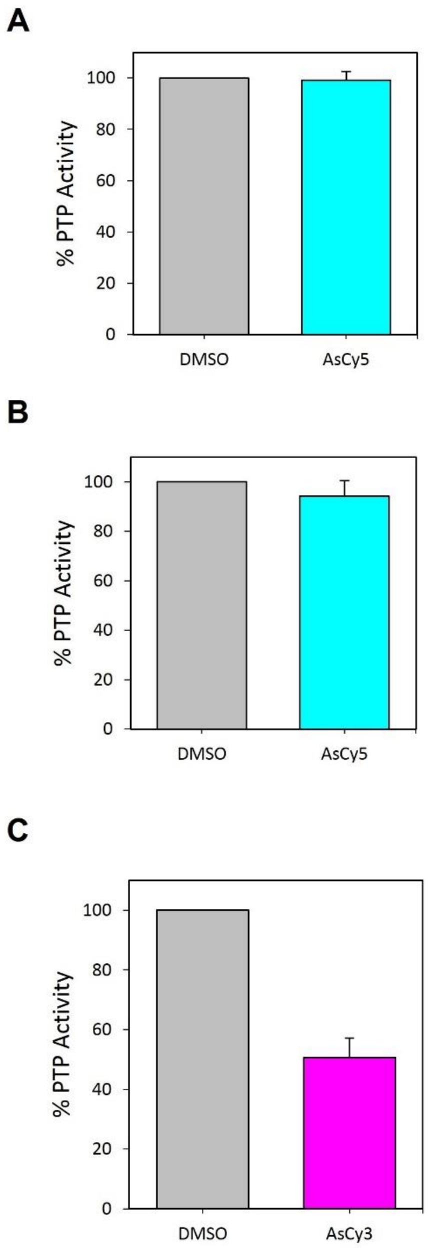

Figure S1. Wild-type PTPs show no activation by biarsenicals. A) PTP activity of wt-HePTP (200 nM) was measured with *p*NPP (1.5 mM, quenched assay) in the absence (DMSO) or presence of AsCy5 (750 nM) after 60-minute pre-incubations. B) PTP activity of wt-PTPκ (400 nM) was measured with *p*NPP (2 mM, quenched assay) in the absence (DMSO) or presence of the indicated biarsenicals (2 µM) after 60-minute pre-incubations. C) PTP activity of wt-SHP2 (50 nM) was measured with *p*NPP (2 mM, quenched assay) in the absence (DMSO) or presence of the indicated biarsenicals (1 µM) after 60-minute pre-incubations.

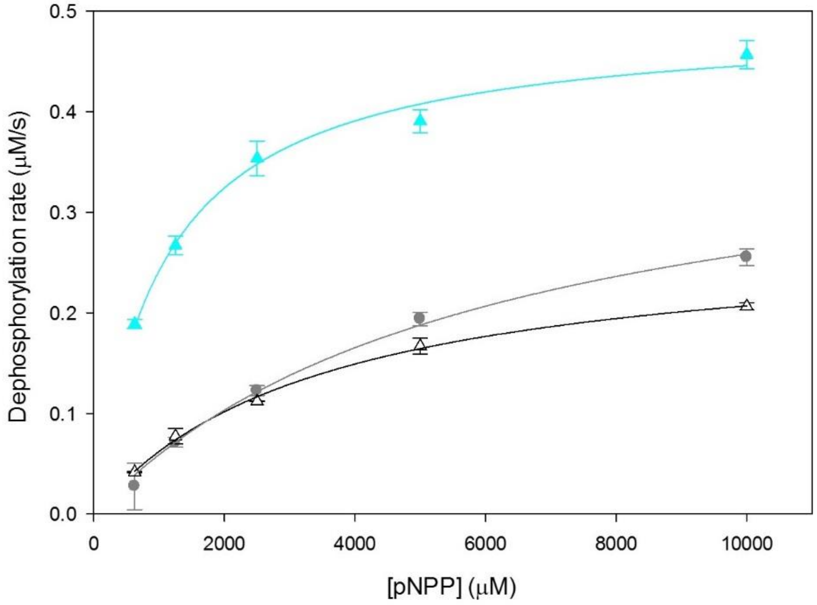

Figure S2. Michaelis-Menten kinetics of wild-type and *act*HePTP. Wild-type (wt) HePTP (200 nM, grey circles: with DMSO) and *act*HePTP (200 nM, white triangles: with DMSO and blue triangles: with AsCy5) were assayed for PTP activity with *p*NPP (indicated concentrations, quenched assay) after 60-min of pre-incubation with AsCy5 (750 nM) or DMSO vehicle. The initial rates of the resulting reactions were fit to the Michaelis-Menten equation to derive the kinetic constants shown in Table 1.

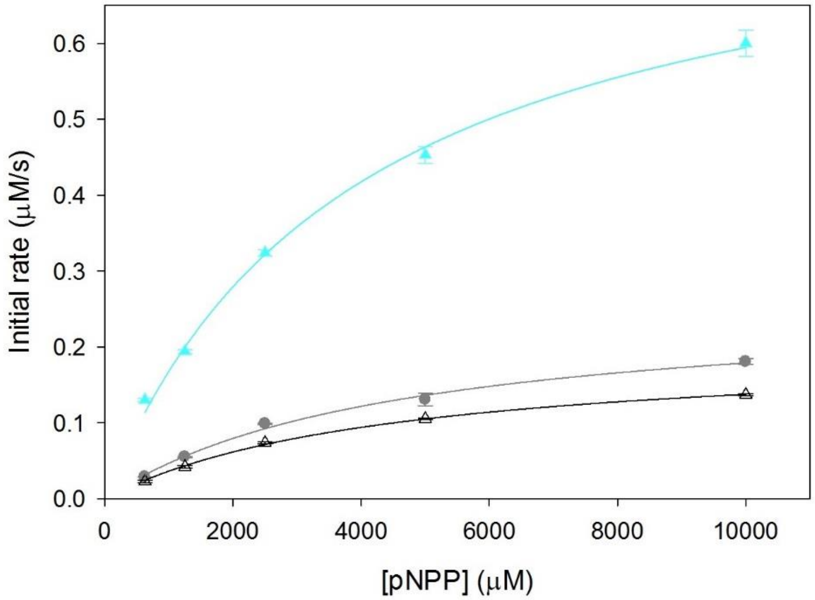

Figure S3. Michaelis-Menten kinetics of wild-type and *act*PTPκ. Wild-type (wt) PTPκ (200 nM, grey circles: with DMSO) and *act*PTPκ (200 nM, white triangles: with DMSO and blue triangles: with AsCy5) were assayed for PTP activity with *p*NPP (indicated concentrations, quenched assay) after 60-min of pre-incubation with AsCy5 (4 μM) or DMSO vehicle. The initial rates of the resulting reactions were fit to the Michaelis-Menten equation to derive the kinetic constants shown in Table 2.

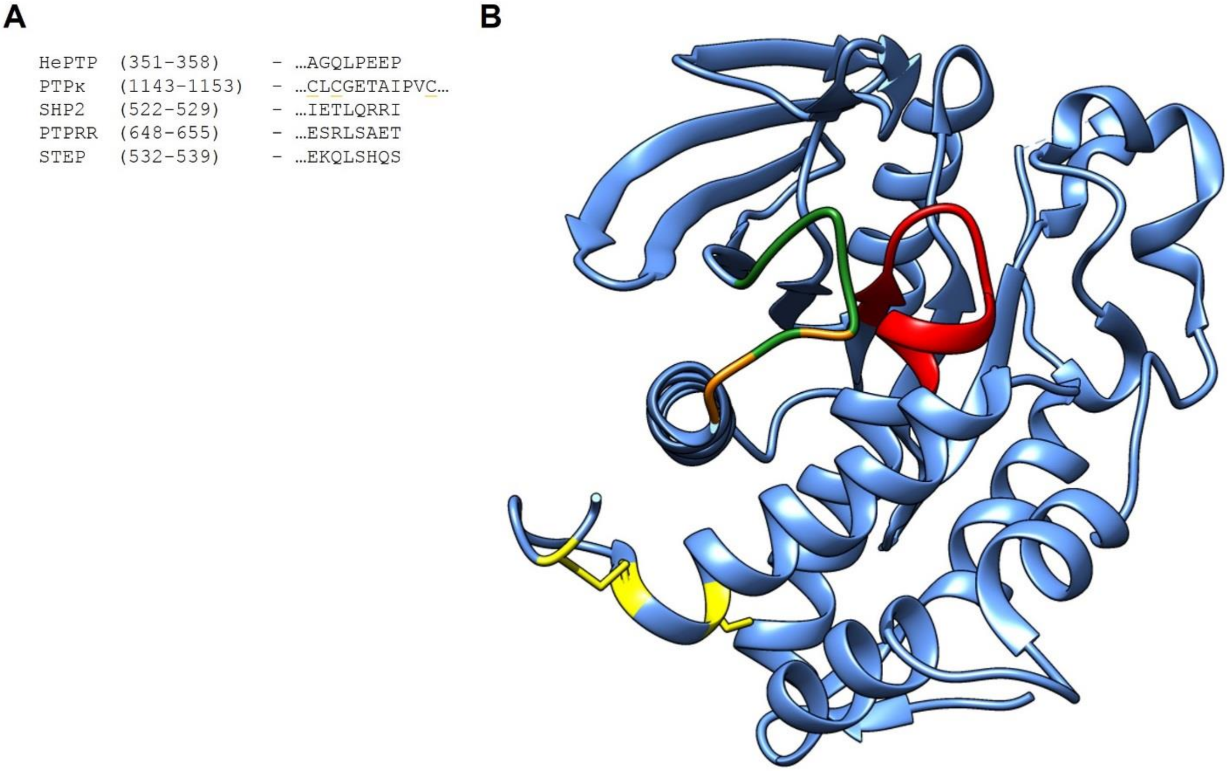

Figure S4. PTPκ contains a nonconserved cysteine-rich region at the C-terminus of the PTP domain. A) Primary sequence alignment of the PTP domain C-terminus of the PTPs investigated in the current study. B) Model of PTPκ. The solved crystal structure of PTPκ (PDB ID: 2C7S) is colored in the UCSF Chimera software package to highlight the catalytic loop (red), the WPD loop (green), the location of the engineered three-cysteine biarsenical tag (orange), and three nonconserved C-terminal cysteine residues (yellow).

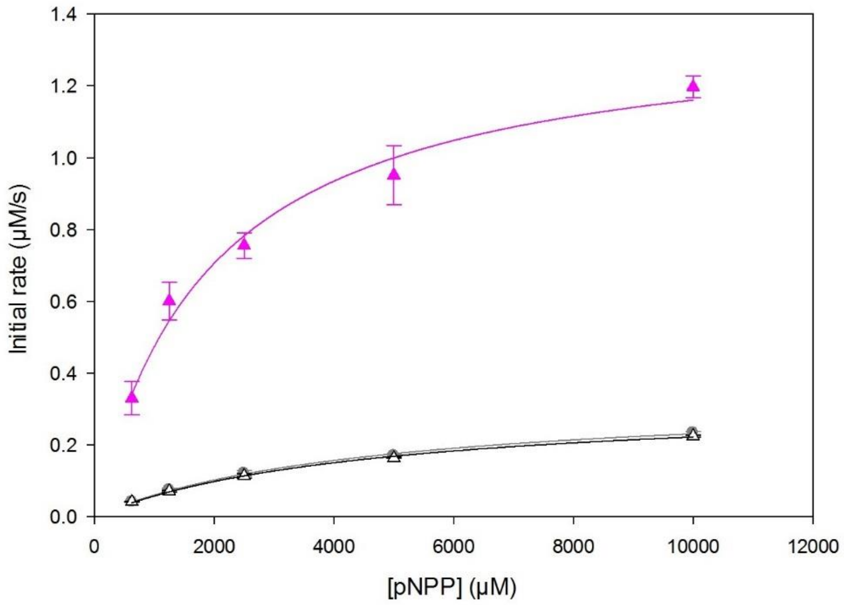

Figure S5. Michaelis-Menten kinetics of wild-type and *act*SHP2. Wild-type (wt) SHP2 (50 nM, grey circles) and *act*SHP2 (50 nM, white triangles: with DMSO and blue triangles: with AsCy5) were assayed for PTP activity with *p*NPP (indicated concentrations, quenched assay) after 15-min of pre-incubation with AsCy3 (1 µM) or DMSO vehicle. The initial rates of the resulting reactions were fit to the Michaelis-Menten equation to derive the kinetic constants shown in Table 3.

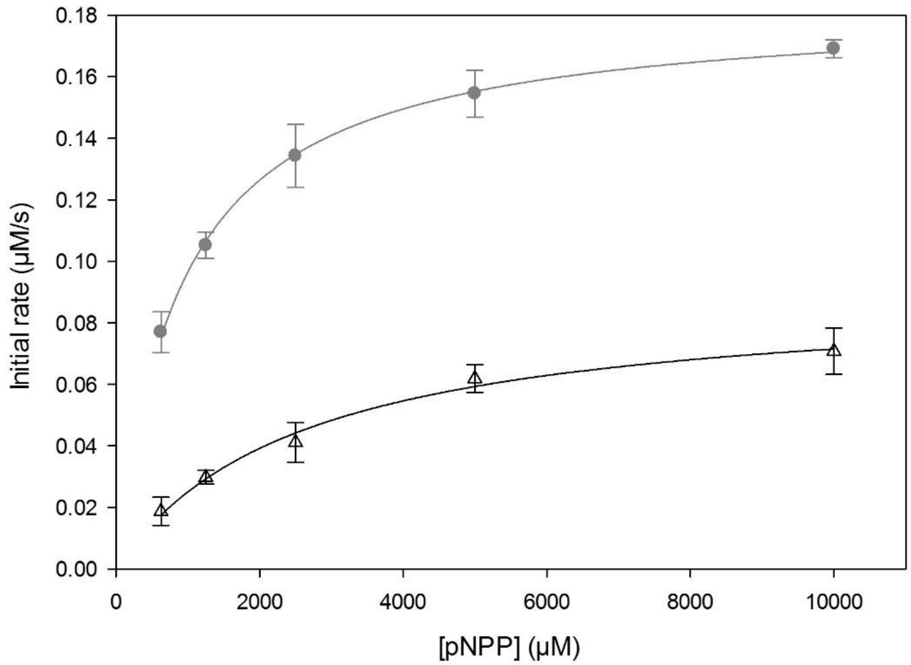

Figure S6. Michaelis-Menten kinetics of wild-type and *act*PTPRR. Wild-type (wt) PTPRR (100 nM, grey circles) and *act*PTPRR (100 nM, white triangles) were assayed for PTP activity with *p*NPP (indicated concentrations, quenched assay). The initial rates of the resulting reactions were fit to the Michaelis-Menten equation to derive the kinetic constants shown in Table 4.

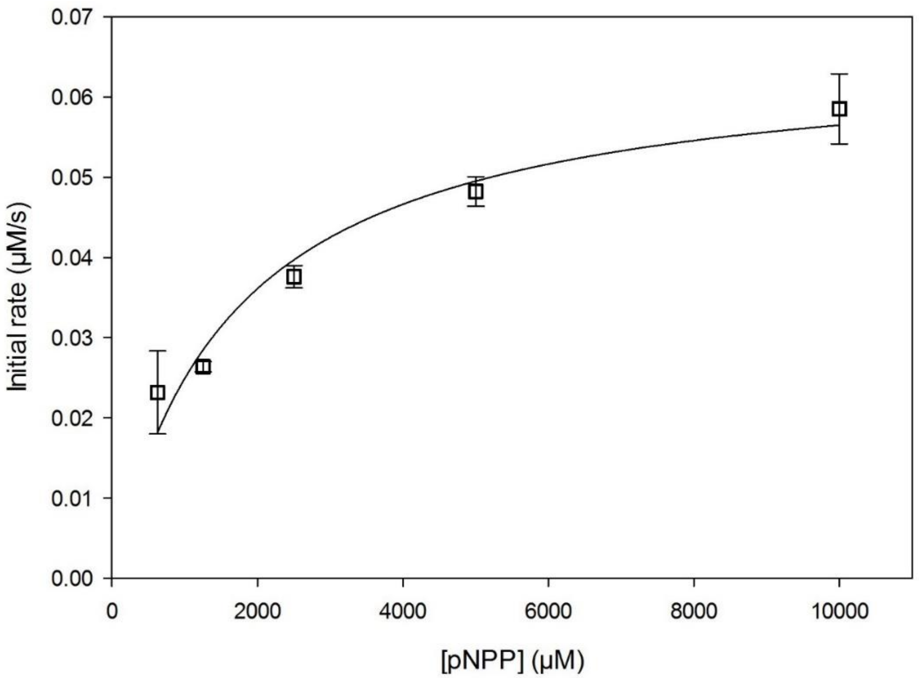

Kinetic constants of K556G *act*PTPRR, assayed with *p*NPP.			
Enzyme	k_{cat} [s^{-1}]	K_M [mM]	k_{cat}/K_M [mM^{-1}s^{-1}]
K556G *act*PTPRR	0.131 ± 0.007	1.6 ± 0.3	0.08 ± 0.02

Figure S7. Michaelis-Menten kinetics of K556G *act*PTPRR. K556G *act*PTPRR (500 nM, white squares) was assayed for PTP activity with *p*NPP (indicated concentrations, quenched assay). The initial rates of the resulting reactions were fit to the Michaelis-Menten equation to derive the kinetic constants shown in the accompanying table.

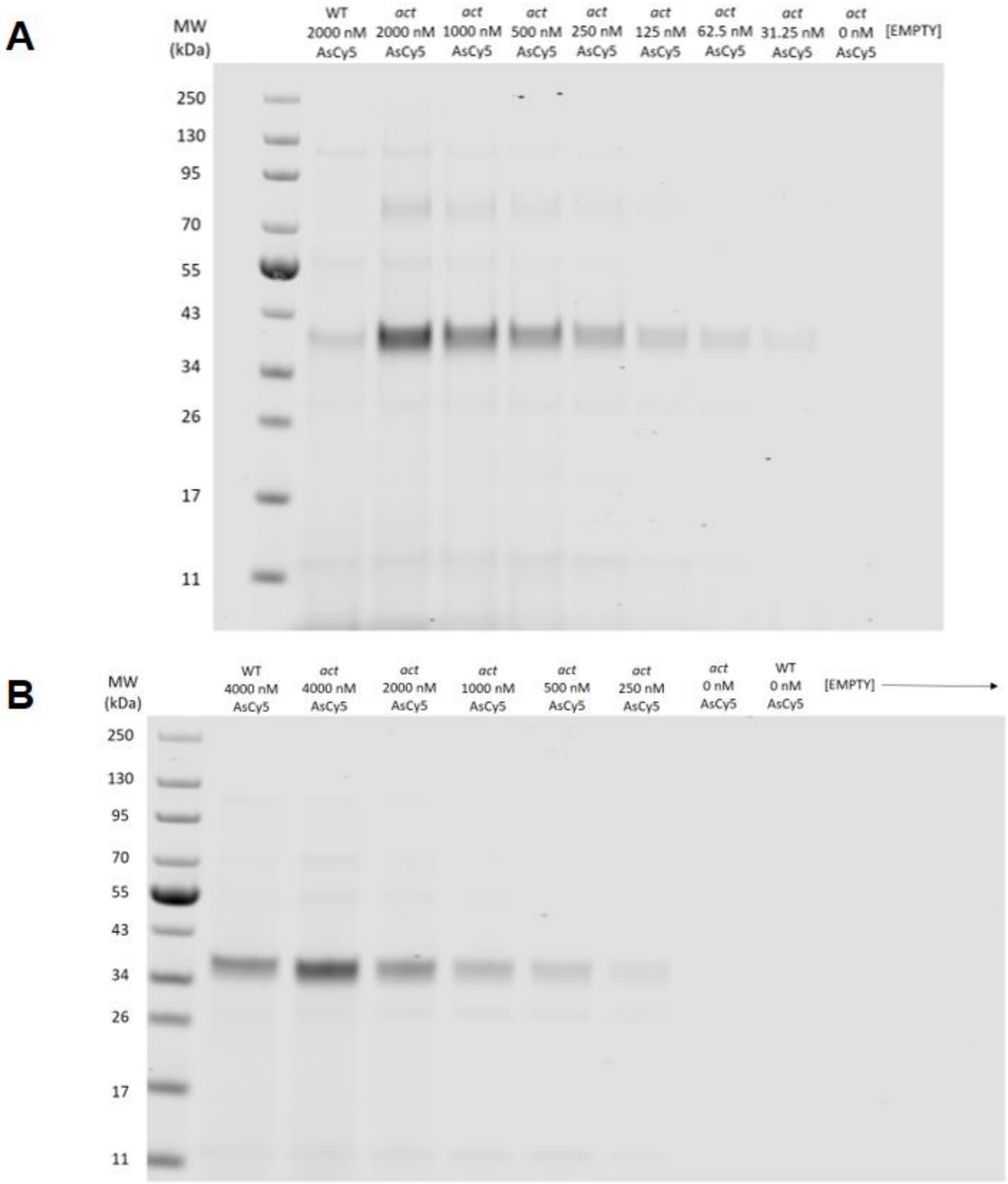

Figure S8. Uncropped images of gels from Figures 3B and 5B. A) Uncropped image of gel from Figure 3B: Clarified lysates (0.085 mg/mL) expressing *act*HePTP or wt-HePTP were incubated with the indicated concentrations of AsCy5 for 15 minutes. The resulting solutions were diluted by a factor of 25, and 15 µL of each dilution were separated by SDS-PAGE. AsCy5-bound proteins were visualized using near infrared illumination. B) Uncropped image of gel from Figure 5B: Clarified lysates (0.125 mg/mL) expressing *act*PTPκ or wt-PTPκ were incubated with the indicated concentrations of AsCy5 for 30 minutes. The resulting solutions were diluted by a factor of 75, and 15 µL of each dilution were separated by SDS-PAGE. AsCy5-bound proteins were visualized using near infrared illumination.
